# Omics Application of Bio-Hydrogen Production Through Green Alga *Chlamydomonas reinhardtii*

**DOI:** 10.3389/fbioe.2019.00201

**Published:** 2019-08-21

**Authors:** Lili Xu, Jianhua Fan, Quanxi Wang

**Affiliations:** ^1^Department of Biology, College of Life Sciences, Shanghai Normal University, Shanghai, China; ^2^State Key Laboratory of South China Sea Marine Resource Utilization, Hainan University, Haikou, China; ^3^State Key Laboratory of Bioreactor Engineering, East China University of Science and Technology, Shanghai, China

**Keywords:** *Chlamydomonas reinhardtii*, hydrogen production, genomics, transcriptomics, proteomics, metabolomics

## Abstract

This article summarizes the current knowledge regarding omics approaches, which include genomics, transcriptomics, proteomics and metabolomics, in the context of bio-hydrogen production in *Chlamydomonas reinhardtii*. In this paper, critical genes (*HydA1, Hyd A2, Sulp, Tla1, Sta7, PFL1*) involved in H_2_ metabolism were identified and analyzed for their function in H_2_ accumulation. Furthermore, the advantages of gene microarrays and RNA-seq were compared, as well as their applications in transcriptomic analysis of H_2_ production. Moreover, as a useful tool, proteomic analysis could identify different proteins that participate in H_2_ metabolism. This review provides fundamental theory and an experimental basis for H_2_ production, and further research effort is needed in this field.

## Introduction

With the increase of the exploitation and utilization of traditional energy, the supply of fossil fuels gradually decreases and the cost of exploiting new energy increases, eventually leading to the gradual rise of energy prices (Hwang et al., 2014; Fakhimi et al., 2019). Traditional energy sources tend to cause environmental problems and air pollution, access to renewable and environmentally sustainable fuels and energy sources may be the greatest challenge of this century. Hydrogen is considered one of the ideal clean-energy alternatives because its only combustion product is H_2_O as well as it has a high heating value (Ramadass et al., 2019). Currently, hydrogen production is expensive, and the energy output hardly exceeds the input. Since Gaffron and his coworkers discovered the presence of hydrogen metabolism in the green algae *Scenedesmus obliquus*, hydrogen biosynthesis from algae attracted many attentions of researchers (Gaffron, 1939; Gaffron and Rubin, 1942). There are many advantages to the use of algae as carrier of hydrogen production, such as the lack of requirement for occupational farmland and the high efficiency of solar energy conversion (Georgianna and Mayfield, 2012).

**Figure 1:**
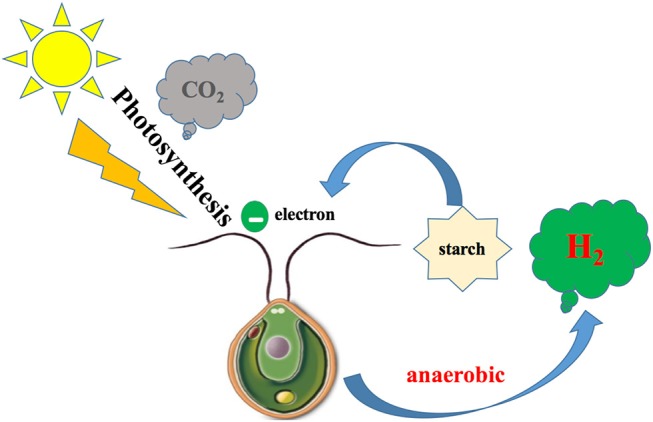
Model diagram of hydrogen production of *C. reinhardtii*.

**Figure 2:**
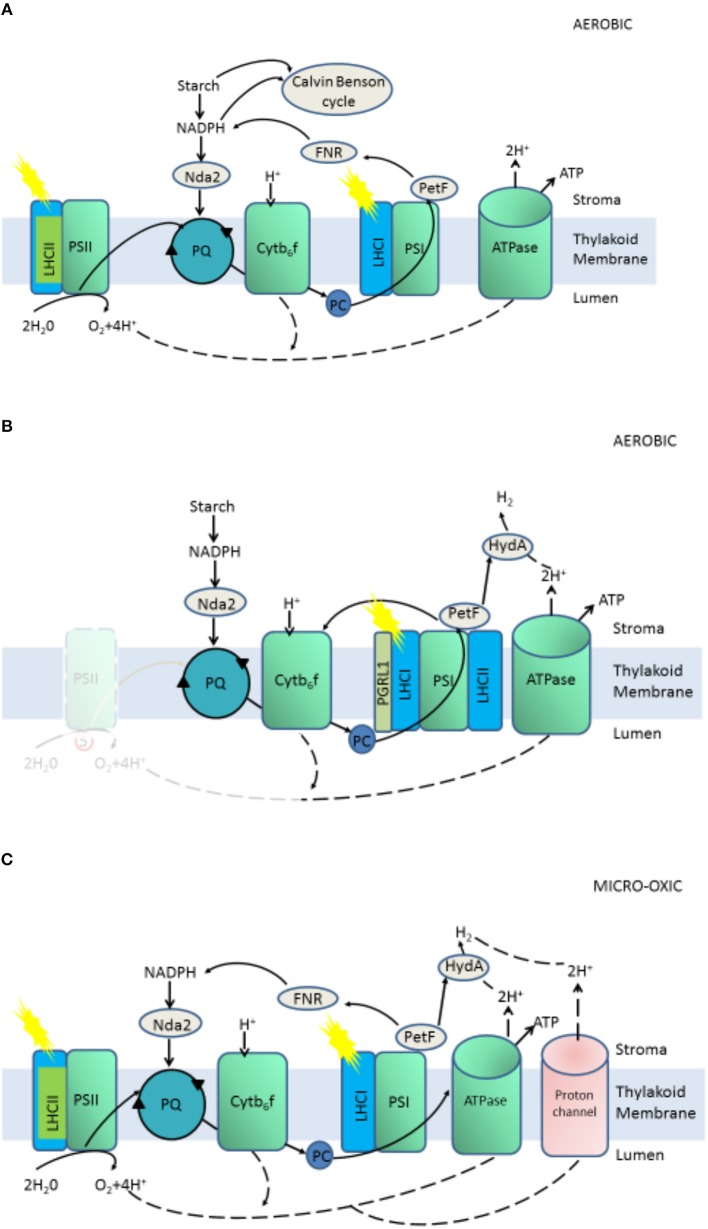
Aerobic **(A)** and anaerobic **(B)** stages of two phases H_2_ production and Micro-oxic continuous H_2_ production **(C)**. Proton(H^+^) flow is marked with dashed lines, electron flow with continuous lines.

*Chlamydomonas reinhardtii*, as a species of unicellular green algae, has been chosen as a model species for studying biohydrogen production because of its sequenced genome; ease of cultivation and low cost; especially, high hydrogenase activity, which catalyzes the reaction of hydrogen formation (Das and Veziroglu, 2001, 2008). The hydrogenase of *C. reinhardtii* could be activated only under anaerobic condition and receive electrons from “photosynthetic electron transport chain” or decomposing intracellular organics, which constitute one-fourth of the photosynthetic electron chain, then reduced to H_2_ and released out of the cell (Melis et al., 2000; Melis, 2007) (Figure 1). The hydrogenase of *C. reinhardtii* is sensitive to oxygen, while oxygen is an inevitable product of photosynthesis, thus the hydrogen production of *C. reinhardtii* in natural state is very low, which is a bottleneck for commercialization of hydrogen production (Roessler and Lein, 1984; Happe and Naber, 1993). To overcome these limitations, some approaches have been used, such as controlling the conditions of cultivation, investigating the structure of hydrogenase to enhance activity, transferring exogenous genes into cells, and screening mutants with high hydrogen yields (Antal et al., 2003; Kruse et al., 2005; Kosourov et al., 2007; Beer et al., 2009; Eroglu and Melis, 2011; Srirangan et al., 2011; Alena et al., 2013; Dubini and Ghirardi, 2015). Low output of hydrogen yield in C*. reinhardtii* under purely anaerobic conditions results in and restricts the commercialization of hydrogen production (Melis et al., 2000; Melis, 2007). Before 2000, Melis proposed that depletion of sulfur from cultures led to sustainable hydrogen production (Melis et al., 2000), which is a landmark discovery for bio-hydrogen production by C*. reinhardtii*. The activity of PSII is inhibited by sulfur deficiency, resulting in a decrease in the rate of oxygen evolution of photosynthesis to rates lower than the rate of oxygen uptake by respiration, and the algal medium changes from an aerobic to anaerobic condition, which activates the hydrogenase (Happe and Naber, 1993; Melis et al., 2000; Antal et al., 2003; Melis, 2007). Hydrogenase-received electrons originate from the photosynthetic electron chain or from decomposing intracellular organics, which constitute one-fourth of the photosynthetic electron chain (Figure 2).

A “two-stage” protocol of hydrogen production proposed by Melis, resulted to oxygen and the production of hydrogen temporally separate by sulfur deficiency (Melis et al., 2000; Melis, 2007). Subsequently, many reports on the hydrogen production of *C. reinhardtii* are based on sulfur deficiency.

Since the human genome has been sequenced, “-omics” techniques used for visualizing entire cell processing has clarified biosynthesis and regulatory networks and developed rapidly. This new technology has created an exciting new frontier for high-throughput, predictable engineering of biofuels. Therefore, considerable attention has been paid to omics analyses of model organism *C. reinhardtii* in different pretreated conditions, which can reveal the molecular and metabolic mechanism comprehensively by three-dimensional analysis (Merchant et al., 2007; Winck et al., 2013; Schmollinger et al., 2014; Smitha et al., 2014; Kleessen et al., 2015; Strenkert et al., 2019). A great number of research regrading omics studies in bio-hydrogen production by *C. reinhardtii* in sulfur-deprived culture have been done. Investigation into the promising production carrier of *C. reinhardtii* and its mutants with these powerful techniques has improved predictability and understanding of the unique, complex interactions within model organisms.

In this paper, we summarize these omics approaches, which include genomic, transcriptomic, proteomic and metabolomics approaches, applied to the biohydrogen production of *C. reinhardtii* in sulfur-deprived culture with the aim of investigating the molecular and mechanism and pathways underlying hydrogen metabolism and regulation at the gene, transcription, protein, and metabolic levels.

## Genomic Analysis of Hydrogen Evolution in *C. reinhardtii*

### Hydrogenases Catalyze Hydrogen Evolution in *C. reinhardtii*

Hydrogen metabolism in *C. reinhardtii* is a complex biochemical reaction catalyzed by hydrogenases, and these enzymes that not only catalyze but also uptake or production of hydrogen, were confirmed to sensitive to O_2_ (Bamberger et al., 1982; Happe and Naber, 1993; Happe et al., 1994; Vignais et al., 2001; Forestier et al., 2003; Boichenko et al., 2004). Hydrogenases are classified into three groups: iron [Fe] only, nickel-iron [Ni-Fe], and no metal ion (Vignais et al., 2001; Boichenko et al., 2004). Hydrogenases of *C. reinhardtii* belong to the [Fe]-hydrogenases group. Isolation and identification of the [Fe]-hydrogenases and their associated genes could improve hydrogen production of *C. reinhardtii* by the manipulation of these genes and proteins. There are two hydrogenases that belong to the [Fe]-hydrogenase group in *C. reinhardtii*. The first of these hydrogenases is termed HydA1 and is encoded by the *HydA1* gene (Forestier et al., 2003). Similarly, the second hydrogenase of *C. reinhardtii* is termed HydA2 and is encoded by the *HydA2* gene. The protein encoded by *HydA1* was 74% similar and 68% identical to HydA2. Both HydA1 and HydA2 contain all motifs and conserved residues which are present in the catalytic cores of the [Fe]-hydrogenase family members. The cDNA of HydA1 and HydA2 have a 158- and 139-nucleotide 5′-UTR, and a 747- and an 873-nucleotide 3′-UTR, respectively (Forestier et al., 2003) (Table 1). The ORFs of *HydA1* gene and *HydA2* gene encode proteins with 497 and 505 amino acid residues, respectively. HydA1 contains 8 exons, while *hydA2* contains 10 exons (Happe and Kaminski, 2002) (Table 1). The analysis of the structure and characteristics of hydrogenase in *C. reinhardtii* provides a theoretical basis for improving the oxygen tolerance by modifying the genetic information, and it will become a promising research direction of hydrogen production.

**Table 1:** Structural characteristics of algal Fe-hydrogenase from *C. reinhardtii* (Forestier et al., 2003).

**Name**	**Exon**	**Intron**	**Coding region(bp)**	**5^**′**^UTR(bp)**	**3^**′**^UTR(bp)**	**ORF**
Hydrogenases (HydA1)	8	7	1,494	158	747	497
Hydrogenases (HydA2)	10	9	1,515	139	873	505

### Genes Associated With Hydrogen Production in *C. reinhardtii*

DNA insertion and nuclear transformation to construct mutants is an effective method for screening novel genes associated with hydrogen production, and the gene families regulated of hydrogen production have been described by many researchers (Table 2).

**Table 2:** Genes are related to hydrogen production in *C. reinhardtii*.

**Gene**	**Mutation**	**Phenotype**	**H_**2**_ production**	**References**
Hydrogenases (HydA) gene	_________	________	Positively related	Melis et al., 2000; Srirangan et al., 2011; Alena et al., 2013
Sulfate permease (Sulp) gene	_________	________	Negatively related	Smitha et al., 2014
Chlorophyll antenna size regulatory genes	Tla1	Higher photosynthetic productivity and greater solar conversion efficiency	Negatively related	Schmollinger et al., 2014
Isoamylase gene	Sta7-10	Insoluble starch in *sta7-10* mutant is less than 5% of the wild type	Positively related	Vignais et al., 2001
Pyruvate formate lyase (PFL1)gene	PFL1	Secretes no formate, but produce more ethanol, D-lactate and CO_2_, the transcript and protein levels of HYD1 and HYD1 were lower than wild type.	Positively related	Ghirardi et al., 2000

#### Sulfate Permease Gene

The production of hydrogen in *C. reinhardtii* could be increased by deficiency of sulfur from cultures and inhibition of the activity of PSII (Melis et al., 2000; Melis, 2007). Therefore, the metabolism of sulfur is closely associated with hydrogen production and it is essential to reveal the sulfur metabolism mechanism and identify genes associated with sulfur metabolism. A novel gene encoded a sulfate permease (*Sulp*) in *C. reinhardtii* was first reported by Chen et al. (2003). The *Sulp* gene of *C. reinhardtii* is nucleus encoded and consists of five exons and four introns, which is different from other *Sulp* genes that are encoded by chloroplasts and lack introns. *SulP* takes an important part in the uptake of sulfur in the chloroplasts of *C. reinhardtii*. RNAi-generated mutants with low levels of *SulP* or lacking *SulP* are expected to be good tools with low rates of H_2_O oxidation but high H_2_ production (Melis et al., 2000; Melis, 2007).

#### Chlorophyll Antenna Size-Regulating Genes

Hydrogenase could accept electrons from the “photosynthetic electron transport chain” and its activity inhibited by the release of oxygen from photosynthesis; therefore, hydrogen metabolism is associated with photosynthesis. Screening photosynthesis regulatory genes is significant and essential for understanding and regulation of hydrogen metabolism in *C. reinhardtii*. The size of the chlorophyll antenna is important for the function of the antenna in photosynthesis. The *Tla1* gene was the first to be identified to regulate the chlorophyll antenna size in photosynthesis and encodes a protein which contains 213 amino acids. The *Tla1* gene-deficient mutant, with a truncated light-harvesting antenna and functional chlorophyll antenna sizes of PSI and PSII of ~65 and 50% that of the control (wild type), respectively, being chlorophyll deficient (Polle et al., 2003). Moreover, the *Tla1* algal strain showed higher photosynthetic productivity and greater solar conversion efficiencies than the control under most culture conditions (Polle et al., 2003). The *Tla1* gene is a promising target to solve the problem of low light utilization efficiency in photosynthesis under biohydrogen production in *C. reinhardtii*. The *Tla1* gene and functionally similar genes will be identified and used in mass cultivation of *C. reinhardtii* for H_2_ production and bioenergy accumulation.

#### Isoamylase Gene

The starch content in *C. reinhardtii* cells is associated with H_2_ production, as starch breakdown could produce the endogenous substrate could donate electrons to the photosynthetic electron chain and mitochondrial electron transport chain, while the electrons of the photosynthetic electron transport chain can supply to hydrogen production (Ghirardi et al., 2000; Melisa and Happe, 2001; Zhang et al., 2002; Kosourov et al., 2003). The *Sta7* gene was identified in a *C. reinhardtii* mutant from a library of 6,000 colonies, and the protein encoded by this gene is similar to the isoamylase enzyme found in other species of plant (Flynn et al., 2002). This enzyme takes an important part in starch metabolism (Dauvillée et al., 2001), and the insoluble starch content in the *sta7-10* mutant is <5% of the wild-type *C. reinhardtii* strain.

The *Sta7* gene may be an important target in research on H_2_ metabolism. It has been shown that hydrogen can be produced continuously as long as the cell contains starch under conditions of sulfur deficiency (Zhang et al., 2002). Consequently, it is hypothesized that the more starch content in the cell, the higher the hydrogen yield of *C. reinhardtii* could be increased accumulation of starch in cells might be achieved by overexpression of the *Sta7* gene of *C. reinhardtii*; thus, increased starch accumulation in *C. reinhardtii* cells could be achieved by normal photosynthesis.

#### Pyruvate Formate Lyase Gene

The procedure of hydrogen metabolism in *C. reinhardtii* is a multiphase process that is not only associated with photosynthesis but also closely associated with dark fermentation (Bamberger et al., 1982). The first step of the dark fermentation reaction is activated by pyruvate lyase (PFL1), which catalyzes pyruvate to form formate and acetyl CoA in this process (Gfeller and Gibbs, 1984; Kreuzberg, 1984; Mus et al., 2007; Hemschemeier et al., 2008). A special *C. reinhardtii* mutant strain 48F5 was screened from library of 5000 colonies. The mutant strain 48F5, in which the affected gene was *PFL1*, does not secrete formate but produces more CO_2_, ethanol and D-lactate than the control, meanwhile the transcription and protein expression levels of HYD1 and HYD1 in mutant strain were lower than those of the control (Philipps et al., 2011). Consequently, the *PFL1* gene may take an important part in H_2_ production related metabolism in *C. reinhardtii*. Additionally, it is hypothesized that increased hydrogen yields might be achieved by overexpression of the *PFL1* gene of *C. reinhardtii*.

## Transcriptomic Analysis of Hydrogen Production in *C. reinhardtii*

### Gene Microarray and RNA-seq

Both gene microarrays and RNA-seq are powerful tools for studying transcription in eucaryons (Table 3). Compared to RNA-seq, gene microarrays are associated with low costs, good coverage of exon-based transcript levels (90%) and low time consumption (Bradford et al., 2010). However, the current microarray platform covers only 87% of the existing genome, and many newly annotated genes have not been identified (Eberhard et al., 2006; Voss et al., 2011). There are expected to be up to 17,000 transcript models in *C. reinhardtii* based on the known genome information (Jain et al., 2007; Prochnik et al., 2010). Therefore, to achieve the same high transcript coverage as RNA-seq, deep sequencing is required. RNA-seq has greater sensitivity for differential gene expression and higher gene coverage than gene microarray (Fu et al., 2009; Wilhelm and Landry, 2009; Feng et al., 2010; Nagalakshmi et al., 2010; Tang et al., 2010; van Vliet, 2010). However, the results and repeatability of RNA-seq are difficult to detect, and good reproducibility is ususlly difficult to obtain. A typical result is that the use of RNA-seq often overestimates the abundance and length-dependent amplification of highly expressed genes (Marioni et al., 2008; Bradford et al., 2010; Liu et al., 2011). These internal challenges associated with data normalization and data analysis clearly should be addressed.

**Table 3:** Advantage and disadvantage of gene microarray and RNA-seq.

	**Advantage**	**Disadvantage**
Gene microarray	Lower costs; less time consuming, good coverage of exon (90%)	Only covers 87% of the predicted genome and many newly annotated genes are missing
RNA-seq	Higher gene coverage	High reproducibility is often difficult to achieve

Some researchers have used gene microarray and RNA-seq approach to analyze the expression levels of genes in *C. reinhardtii* with sulfur-deprived H_2_ production conditions. Transcriptional analysis of *C. reinhardtii* by the microarray approach was first reported by Nguyen et al. Finally, 166 markedly differentially expressed genes were identified, and these genes were divided into different functional groups by genome comparison: 22 genes related to in photosynthesis, 8 genes related to sulfur metabolism, 4 genes related to carbon metabolism, 5 genes related to proteolysis, 4 genes related to amino acid synthesis, 10 genes were related to transcription and translation, 3 genes related to redox cycling, 27 genes were involved in other processes and pathways, functions of 83 genes were unknown (Nguyen et al., 2008). This study provides a novel insight for the expression and regulation of genes involved in sulfur metabolism, photosynthesis and carbon metabolism in the process of sulfur-deficient hydrogen production.

Toepel et al. analyzed the transcript levels of *C. reinhardtii* in nitrogen- and sulfate-limited cultures by the new microarray method, and 813 downregulated genes and 100 upregulated genes were identified under sulfur-deprived conditions (Toepel et al., 2011). The new microarray data were highly similar to those for many genes in terms of changes in differential gene expression patterns between the first-generation conditions and sulfur-deprived conditions (Toepel et al., 2013). The author of this paper stated that microarrays are inexpensive and reliable tools for detecting the expression levels of changes in transcription, although RNA-seq analysis provide detailed information regarding the transcriptome, have low costs, and exhibit good reproducibility; moreover, this work provides a new microarray platform for transcriptome analysis of *C. reinhardtii* and the established analysis systems can be used with microarrays for routine applications.

RNA-seq technology was used to detect the response of hydrogen production of *C. reinhardtii* cells and mutant cells by Authur's group under sulfur-deficient conditions. Sulfur- deficient *C. reinhardtii* cells gathering transcripts involved in the synthesis of sulfur-containing metabolites, sulfur acquisition, sulfur assimilation, sulfur recycling, and Cys degradation (González-Ballester et al., 2010). Furthermore, changes in cellular structures could occur during the process of sulfur deprivation, including the structure of photosynthetic complexes and the cell wall (González-Ballester et al., 2010). Additionally, this research shows that the protein accumulated by the cells under sulfur deficiency conditions has less sulfur content (González-Ballester et al., 2010).

Since most of the sulfur-deficient reactions are controlled by the SNRK2.1 protein kinase, the mutant exhibits a number of reactions different from those of wild-type cells under conditions of sulfur-deficient and hydrogen production. The mutant strain could not adapt to the increased oxygen content and oxidative stress caused by sulfur deficiency, which led to cell death (González-Ballester et al., 2010). The results of transcriptome analysis showed that the mutants and wild algae have significant changes in the physiology and metabolism under the condition of sulfur deficiency and hydrogen production. This change is important for maintaining the survival of cells under sulfur deficiency stress.

### MicroRNA (miRNA)-seq

MicroRNA (miRNAs) regulates many important metabolic processes in eukaryotes Hu's group has identified expression level of changes in miRNA through a deep sequencing platform and extensively profile in the process of H_2_ production and sulfur-deficient culture (Shu and Hu, 2012). The results indicated that sulfur-deprived conditions may have an appreciable impact on miRNA expression patterns. In this study, the expression levels of 47 miRNAs were significantly different under sulfur-deprived conditions; meanwhile, 310 miRNAs, including 225 novel miRNAs and 85 known miRNAs, were predicted and analyzed. In particular, 13 miRNAs were closely associated with the response to sulfur-deprived conditions; moreover, based on the published information of the transcriptome, target gene associated with metabolic response to the deficiency of sulfur stress were identified in *C. reinhardtii* (Shu and Hu, 2012). The differential interactions and expression of miRNAs and their potential targets could reveal the molecular mechanism of hydrogen metabolism response to sulfur deprivation in *C. reinhardtii*.

### Genome-wide Long Non-coding RNA (IncRNA) Screening and Characterization

A considerable portion of the genome of eukaryotes can be transcribed to RNAs, but will not be translated to proteins. These non-coding RNAs (ncRNAs) consist of housekeeping, regulatory and functional unknown ncRNAs. Regulatory ncRNAs are usually classified as small non-coding RNAs and long non-coding RNAs (lncRNAs) according to their lengths (Shu and Hu, 2010; Pauli et al., 2011). lncRNAs play important roles in cell differentiation and development (Guttman et al., 2011; Pauli et al., 2011; Fatica and Bozzoni, 2014) silencing gene expression in X-chromosome (Dimond and Fraser, 2013), neurological diseases occurrence (Ponting et al., 2009), cancer progression (Wapinski and Chang, 2011; Yang et al., 2014) and immune response genes mediation (Carpenter et al., 2013; Wang et al., 2016). In plants, lncRNAs express differentially in various organs and under different treatment conditions, which indicates lncRNAs can modulate gene activity during development and in response to external stimuli (Kim and Sung, 2012). Based on the regulatory functions found in other higher plants, lncRNA investigation and manipulation may provide new insights and solutions for green algae. Transcriptome and proteome analyses indicated that sulfur deprivation affects massive pathways including sulfur metabolism, cell wall structure, photosystems, protein biosynthetic apparatus, molecular chaperones and 20 S proteasomal components (Nguyen et al., 2008; Chen et al., 2010; González-Ballester et al., 2010). It was previously demonstrated that hydrogen production can be regulated by an artificial non-coding RNA miRNA (amiRNA) targeting OEE2 encoded gene (a photosystem II related protein, oxygen evolving enhancer) (Li et al., 2015). RNA sequencing in *C. reinhardtii* under hydrogen production and sulfur deficient condition has been done and obtained totally 3,574 putative lncRNAs. 1440 were considered as high-confidence lncRNAs, including 936 large intergenic, 310 intronic and 194 anti-sense lncRNAs. The average transcript length, ORF length and numbers of exons for lncRNAs are much less than for genes in this green algae (Li et al., 2016). In addition, 367 lncRNAs responsive to sulfur deprivation was identified, including 36 photosynthesis-related lncRNAs (Li et al., 2016). lncRNAs used to reveal the molecular and metabolism mechanisms in *C. reinhardtii* are very rare, nevertheless the lncRNA data could provide new insights into *C. reinhardtii* hydrogen production under sulfur deprivation.

## Proteomic Analysis of H_2_ Photoproduction in *C. reinhardtii*

Since the completion of *C. reinhardtii* genome sequencing and annotation (Merchant et al., 2007), the research of *C. reinhardtii* has opened up a new era. DNA-microarray analysis and transcriptome analysis indicated that more than 100 genes were up- or down-regulated throughout the process of sulfur-depleted H_2_ photoproduction (Nguyen et al., 2008). These findings had revealed the mechanism of H_2_ metabolism in *C. reinhardtii* at the genetic level. Meanwhile proteomic analysis has been regarded as a powerful tool to study global translational profiles for biological processes. At the protein level, however, experimental data about H_2_ metabolism in *C. reinhardtii* are still limited. Comparative proteomics to assess the expression level of proteomic changes in *C. reinhardtii* under sulfur-depleted H_2_ released conditions has been achieved (Chen et al., 2010). Total 159 different protein spots were identified, 105 were found enhanced or reduced significantly, corresponding to 82 unique genes, throughout H_2_ production under sulfur deprivation. Meanwhile the photosynthetic machinery, molecular chaperones and protein biosynthetic apparatus were changed significantly in the process. Additionally, many proteins associated with anti-oxidative reactions and sulfate, acetate, and nitrogen metabolism also changed markedly. Furthermore, other proteins involved in cell wall and flagellum metabolism showed changes under sulfur-depleted H_2_ released conditions. These data provide not only detailed information regarding the complex interactions between photosynthesis and hydrogen metabolism in switching the organism from O_2_-Generating to H_2_ production, but also more candidate genes for targeted genetic engineering of *C. reinhardtii* that would lead to further elucidation of the mechanisms of H_2_ production and its large-scale utilization.

## Metabolomics Analysis of H_2_ Photoproduction in *C. reinhardtii*

The understanding of the metabolic pathways essential to the hydrogen production metabolism is fundamental to identify metabolites and proteins which may relate to the cell acclimation to environmental changes. Based on the genome sequence it is possible to develop models of the metabolic network for some organisms and to predict their behavior under defined conditions. Metabolic network models may serve as a basis for in hydrogen metabolic engineering, and their complementation with experimental data will certainly improve the prediction capacity of the available models (Pauli et al., 2011). In order to elucidate the role of the metabolic pathways and metabolites into the biological systems, theoretical and experimental approaches have been performed. Single metabolite analysis has not been reported, metabolomics analysis of H_2_ photoproduction in *C. reinhardtii* were combined with transcriptomic and proteomics analysis (May et al., 2008; Subramanian et al., 2014).

## Application of Multiomics Technique in H_2_ Photoproduction by *C. reinhardtii*

The unique complexity and adaptability of *C. reinhardtii* to maintain its cellular activity under H_2_ production and sulfur deficiency conditions is well-known. H_2_ production metabolism, fermentative pathways and photosynthesis become active during the process (May et al., 2008; Subramanian et al., 2014). A combination of multiple omics techniques, transcriptomics, proteomics and metabolomics analyses, is a useful tool to study H_2_ metabolism in *C. reinhardtii* under sulfur-depleted H_2_ released conditions. Up-regulated proteins and down-regulated proteins were detected by transcriptomics and proteomics analysis and those proteins belong to flagellum proteins, light-harvesting complex proteins, glyoxylate cycle proteins, nitrogen reorganization proteins, putative redox proteins and ATP synthase proteins by functional annotation (Subramanian et al., 2014). Additionally, combined with the analysis of metabolites by GC-MS, strong evidence showed that the glyoxylate pathway could reverse TCA reactions, which helps in conserving carbon within the cell, while simultaneously reoxidizing NADH. Meanwhile, the presence of the serine-isocitrate lyase pathway has been reported to be active in this process. Finally, *C. reinhardtii* appears to cope with the reduced cellular energy levels under sulfur-depleted H_2_ released conditions, by relying on glycolysis and fermentation in order to generate more ATP and regenerate NAD^+^, respectively, for continuity of catabolic processes. Future work would require measurement of energy allocation to different metabolite pathways within the cell over longer periods of sulfur-depleted H_2_ production, and understanding the alteration of the metabolism in sulfur-depleted H_2_ production conditions.

## Conclusions and Prospects

H_2_ production by *C. reinhardtii* may provide an effective method to solve the energy crisis and is a complex physiological process involved in photosynthesis, respiration, and fermentation. Many researchers have studied this physiological process. Currently, omics technologies (genomics, transcriptomics, proteomics, and metabolomics) have applications in the bioproduction of H_2_ in *C. reinhardtii* and could be used to comprehensively investigate the underlying mechanisms, leading to the utilization of this process on an industrial scale.

## Author Contributions

LX and QW conceived and designed the paper. LX and JF wrote and revised the paper.

### Conflict of Interest Statement

The authors declare that the research was conducted in the absence of any commercial or financial relationships that could be construed as a potential conflict of interest.
